# Maternal attitudes and practices toward childhood fever: insights from a large-scale survey of over 3,000 mothers

**DOI:** 10.1186/s12887-025-06335-8

**Published:** 2025-11-24

**Authors:** Mehmet Cengiz, Bahar Öztelcan Gündüz

**Affiliations:** 1 Department of Pediatrics, Özel Gürlife Hospital, Aşağısöğütönü mah. 1536.Sk. Bina No:1, Beril apt. Kat: 2 No: 4 Tepebaşı, Eskişehir, Turkey; 2 Department of Pediatrics, University of Health Sciences, Gülhane Training and Research Hospital, Ankara, Turkey

**Keywords:** Childhood fever, Parental knowledge, Fever management, Febrile seizures, Antipyretic use, Parental anxiety

## Abstract

**Background:**

Fever is a common childhood symptom and a frequent source of parental concern. Despite medical advances, misconceptions and inappropriate management strategies remain widespread. This study aimed to describe maternal knowledge, attitudes, and practices regarding childhood fever in Türkiye and to identify areas that may benefit from targeted education.

**Methods:**

We conducted a cross-sectional, online survey using snowball sampling. A total of 3,133 mothers provided complete responses. The questionnaire assessed thermometer use, fever definitions, attitudes, and management practices. Descriptive statistics were reported, with exploratory comparisons conducted where relevant.

**Results:**

The mean age of the participants was 33.4 ± 6.0 years, and the average age at which they became mothers was 27.6 ± 6.7 years. Digital thermometers were the most commonly used devices (83.1%), while 13% reported use of mercury thermometers, likely reflecting legacy devices or misclassification. Fever was most frequently defined as ≥ 38 °C (42.7%). Parental anxiety scores were high (mean 7.7/10), with febrile seizures being the most feared complication (73.1%). Antipyretics were often given early and at short intervals, with 39% of mothers administering them every 4 h or less. While most mothers relied on physicians for information, some used unvalidated practices such as vinegar or alcohol rubs.

**Conclusions:**

This large descriptive survey suggests that fever phobia and misconceptions persist among mothers in Türkiye. Findings should be interpreted cautiously due to the study’s design limitations, including non-representative sampling and lack of child-level age data. These results provide a baseline for future, representative studies and may help inform parental counseling strategies.

**Supplementary Information:**

The online version contains supplementary material available at 10.1186/s12887-025-06335-8.

## Introduction

Fever is a physiological response regulated by the hypothalamic region of the central nervous system, occurring when body temperature rises above its normal range due to various factors [1]. Although fever is commonly associated with benign, self-limiting illnesses, it often causes significant anxiety among parents [2]. While healthcare professionals recognize fever as a natural defense mechanism, studies indicate that parents frequently resort to antipyretic medications, even in non-critical situations [3]. In some cases, concerns persist that fever could lead to neurological damage, seizures, or even death [4]. Such fears are further exacerbated by the common pediatric practice of alternating different antipyretics in febrile conditions [5]. Consequently, parental anxiety may contribute to inappropriate fever management, including excessive dosing, unnecessary use of antipyretics at normal body temperatures, and reliance on traditional but ineffective methods such as alcohol rubs, vinegar applications, or cold baths [6, 7]. Parental approaches to fever management are influenced by multiple factors, including education level, prior experiences, and socioeconomic background [8].

This study was designed as a descriptive, exploratory survey to characterize parental knowledge, attitudes, and practices regarding childhood fever in Türkiye. Our a priori analytic objective was to provide population-level descriptive estimates of thermometer use, fever definitions, information sources, antipyretic practices, and fever-related concerns. Any between-group comparisons (e.g., by maternal education or income) were post hoc exploratory and are presented as hypothesis-generating rather than confirmatory.

## Methods

### Sampling and recruitment endpoint

We used snowball sampling via social media to maximize reach within a fixed recruitment window (start–end dates). Data collection ceased when the pre-specified time window ended and responses stabilized operationally (no active recruitment channels remained and daily accrual fell to a minimal trickle). We did not perform a formal sample-size calculation because our primary objective was descriptive estimation; thus, precision is reflected in the reported confidence intervals and narrow standard errors around key proportions. We acknowledge that snowball sampling may limit representativeness and can over-sample certain socio-demographic strata.

### Data collection

Data were collected using an online survey distributed via social media platforms, employing a snowball sampling method. The survey included questions regarding parental knowledge, attitudes, and behaviors related to childhood fever management. The questionnaire used in this study was developed by the authors based on a synthesis of items from previous literature on parental fever knowledge and management practices. An English version of the questionnaire is provided as Supplementary File 1.

### Statistical analysis

All responses were anonymized, and informed consent was obtained from participants before data collection. The collected data were analyzed using IBM SPSS Statistics version 29.0 (IBM Corp., Armonk, NY, USA). Descriptive statistics were used to summarize the data, with categorical variables presented as frequencies and percentages, and continuous variables expressed as mean ± standard deviation. Group comparisons were performed using the chi-square test for categorical variables and the Student’s t-test for continuous variables.

We summarized categorical variables as counts (%) and continuous variables as mean ± SD, consistent with our descriptive, exploratory aim, we emphasize descriptive estimates. Any groupwise comparisons are labeled exploratory and interpreted cautiously. We did not conduct confirmatory multivariable modeling; where applicable, we report exploratory associations only to inform future hypothesis-driven studies.

## Results

A total of 3,170 parents participated in the study, of whom 3,133 completed the survey in full and were included in the final analysis. The mean age of participants was 33.4 ± 6.0 years, while the mean age at which they became mothers was 27.6 ± 6.7 years. Regarding the number of children, 52.6% (*n* = 1,648) of respondents had only one child, 36.6% (*n* = 1,147) had two children, 8.6% (*n* = 270) had three children, and 2.2% (*n* = 68) had four or more children. The majority of participants (88.2%) reported that their children did not have any known chronic illnesses.

In terms of educational background, 66.6% of respondents had a university degree or higher, while 24.8% had completed high school, 5.3% had completed middle school, and 3.3% had only a primary school education. Nearly half of the mothers (49.9%) were homemakers. Among working mothers, the most common professions were teaching (13.7%), healthcare (9.3%), civil service (5.0%), and other professions (20.1%). Regarding household income, 57.4% of families reported an income above 10,000 TL, 31.0% reported an income between 5,500 and 10,000 TL, and 11.6% reported an income below 5,500 TL.

When asked about fever measurement practices, 83.1% of mothers reported using a digital thermometer to measure body temperature, primarily on the skin surface. Notably, 13% of respondents reported using mercury thermometers. Because mercury thermometers are not recommended and are uncommon in current practice, this figure may reflect legacy household devices or misclassification (e.g., alcohol-based glass thermometers). We therefore interpret this as a measurement limitation rather than current standard practice.

Regarding the preferred site for temperature measurement, 33.6% of respondents used the axillary (underarm) region, 15.9% used the ear, and 49.9% used other areas such as the forehead, neck, or temples. Only 0.3% reported measuring fever rectally, and 0.2% measured it orally.

When asked at what temperature they considered a child to have a fever, 42.7% of mothers responded ≥ 38 °C, 29.5% responded ≥ 37.5 °C, 17.8% responded ≥ 38.5 °C, and 6.5% responded ≥ 37 °C.

In terms of initial fever management strategies, the most commonly reported actions included removing clothing (63.2%), administering an antipyretic syrup (21.1%), and giving a lukewarm bath (14.7%). Less frequently reported practices included wiping the child’s body with vinegar or alcohol-based solutions, bathing the child in cold water, or administering antibiotics for fever reduction.

When asked about their biggest concerns related to fever, 73.1% of mothers feared febrile seizures, 12.0% were concerned about potential brain damage, and 7.7% perceived fever as a sign of a serious illness. Less frequently mentioned concerns included dehydration, coma, and death.

A vast majority (97.1%) of mothers correctly stated that antibiotics should not be used to reduce fever. However, 2.9% believed that antibiotics could serve as an antipyretic, highlighting a significant misconception.

Regarding antipyretic medication use, 87.8% of mothers reported administering paracetamol-based medications, while 33.6% used ibuprofen-based medications. Only 0.6% reported using antibiotics for fever reduction. Other medications, including aspirin, corticosteroids, and metamizole (novalgin), were used at lower frequencies.

When asked about the dosing intervals of antipyretics, 57.3% of mothers reported administering them every 6 h, while 39.0% administered them every 4 h. A small proportion reported shorter intervals: 2.7% every 3 h, 0.9% every 2 h, and 0.2% every hour. Antipyretics were administered orally in 98.8% of cases, while 1.2% of mothers reported using the rectal route.

Regarding the sources of information on fever management, 72.2% of mothers reported that they obtained their information from physicians, while 14.5% relied on the internet, television, or social media, and 8.8% cited friends, family, or acquaintances as sources. Additionally, 4.5% of respondents reported receiving information from nurses or midwives.

Mothers reported checking their child’s temperature every 15 min (41.0%), every 30 min (40.6%), or every hour (15.8%) when fever was present. When asked about how they determined the dosage of antipyretic medications, 83.3% reported following the measurement provided with the medication, while 15.9% relied on household utensils such as teaspoons or tablespoons.

In terms of antipyretic administration based on recommendations, 70.9% of mothers followed a physician’s advice, whereas 26.9% relied on their own experience. A small proportion reported administering antipyretics based on recommendations from neighbors, pharmacists, nurses, or midwives.

A total of 23.3% of mothers reported administering an antipyretic after their child received a vaccine, while 0.9% admitted to using antibiotics without a prescription. When asked to rate their level of anxiety about fever on a scale from 1 (lowest) to 10 (highest), the mean anxiety score was 7.7 ± 2.1.

## Discussion

Our findings reaffirm persistent “fever phobia,” highlight variability in home measurement and dosing practices, and identify actionable targets for parental education. However, the snowball sampling, lack of regional stratification, and absence of child-level age data limit generalizability and preclude age-specific inferences about management behaviors. Accordingly, we frame these results as nationally informative but not nationally representative.

Our results align with previous studies in the literature. The concept of “fever phobia” was first introduced by Schmitt in 1980 [9], and subsequent research has consistently demonstrated that parents tend to overestimate the dangers of fever, leading to mismanagement strategies [5, 10]. A study conducted in Germany highlighted that parental fear of fever is widespread and often reinforced by misconceptions about its association with neurological damage and febrile seizures [11]. Similarly, a study in Lebanon reported that many parents lacked adequate knowledge about fever management and frequently sought unnecessary medical attention in an attempt to control fever [12]. Consistent with these findings, our study revealed that a large proportion of mothers sought medical care immediately when their child developed a fever, contributing to an increased and often unnecessary burden on healthcare systems.

Misuse of antipyretics was another common issue observed in our study. Excessive administration of fever-reducing medications and alternating between different types of antipyretics have also been reported in previous research [13, 14]. Himbert et al. [11] found that parents frequently administered antipyretics without considering clinical indications, aiming to reduce fever to normal levels rather than focusing on the child’s comfort. Our findings similarly indicate that many mothers administered antipyretics without medical necessity, often giving excessive doses or waking their children at night to provide medication. These behaviors reflect a fundamental misunderstanding of fever as a natural immune response rather than a condition requiring aggressive intervention. Greater emphasis on parental education is needed to reinforce the evidence-based approach that antipyretics should be used primarily for symptom relief rather than for achieving normothermia.

The persistence of non-scientific, traditional fever management practices was also evident in our findings. Studies conducted in different cultural contexts have shown that parents frequently rely on traditional remedies to lower fever [15]. For example, in Southeast Asia, some parents use “talisman water”, in China, herbal remedies are common, while in Japan, warming the child’s body is a popular practice for managing fever [15]. Our study similarly revealed that some mothers opted for ineffective and potentially harmful interventions such as wiping the child’s body with vinegar or alcohol-based solutions. These findings underscore the role of cultural beliefs in shaping fever management practices and highlight the need for healthcare professionals to provide culturally sensitive education when addressing parental concerns. Furthermore, some parents reported using cold showers or ice packs to lower fever, which can result in abrupt changes in body temperature and exacerbate discomfort. These findings emphasize the importance of expanding parental education initiatives beyond medication use to include guidance on scientifically validated and safe fever management strategies.

Misconceptions and fears regarding febrile seizures were another prominent finding in our study. Alawwadh et al. [16] reported that many parents mistakenly equate febrile seizures with epilepsy, leading to heightened anxiety and unnecessary emergency visits. Our results similarly indicate that mothers perceive febrile seizures as a severe medical threat, prompting immediate healthcare utilization whenever their child develops a fever. To address these concerns, healthcare providers must engage in more comprehensive education efforts, offering clear guidance on febrile seizure management and providing reassurance to parents. Additionally, incorrect parental interventions during febrile seizures were noted. Some parents believed that placing objects in the child’s mouth or shaking them vigorously were appropriate responses, despite the potential for causing harm. Educating parents about the correct response to febrile seizures is crucial for ensuring child safety and preventing unnecessary panic-driven actions.

This study has several limitations. First, the **snowball**,** online** recruitment strategy may introduce selection bias and limits representativeness. Second, we did **not collect child-level age** and other key characteristics that are known to shape fever management; thus, age-specific inferences cannot be made. Third, thermometer type may be **misclassified** (e.g., legacy glass devices reported as “mercury”), so those results should be interpreted cautiously. Fourth, the anxiety score was measured by a **single 10-point item**; while practical, it lacks the psychometric depth of validated multi-item scales. Finally, self-report may not fully reflect real-world behaviors.

In addition to the limitations already noted, two issues require further emphasis. First, the absence of child-level data—particularly age—represents a critical limitation, as fever management practices and parental anxiety are strongly age-dependent. Second, while our large sample provides valuable insights, the snowball sampling approach, lack of regional stratification, and absence of multivariable adjustment substantially restrict the generalizability of our findings. Therefore, results should be considered hypothesis-generating rather than nationally representative. Future prospective studies are needed to assess how parental knowledge and behaviors evolve with targeted educational interventions.

## Conclusion

This large descriptive survey identifies prevalent misconceptions and suboptimal practices around childhood fever and underscores the need for targeted, culturally sensitive education for parents. Given design limitations, we avoid policy-level claims; instead, we propose these estimates as a baseline for future, representative, age-stratified studies and as practical signposts for clinician-delivered counseling.

## Supplementary Information

Supplementary material 1.

## Data Availability

The datasets generated and analyzed during the current study are available from the corresponding author on reasonable request.

## Abbreviations

CI: Confidence Interval
IBM: International business machines
SPSS: Statistical package for the social sciences
KAP: Knowledge, attitudes, and practices
SD: Standard deviation
TL: Turkish lira
WHO: World Health Organization
