# Supplementary material for: Maternal attitudes and practices toward childhood fever: insights from a large-scale survey of over 3,000 mothers
Source: BMC Pediatr. 2025 Nov 24;25:947. doi: 10.1186/s12887-025-06335-8 (PMC12642302; doi:10.1186/s12887-025-06335-8)
Supplement: Supplementary file 1 — Supplementary material 1. [file 12887_2025_6335_MOESM1_ESM.pdf]

Supplementary File 1: English Translation of the Questionnaire Used in the Study

---

MATERNAL ATTITUDES AND PRACTICES

TOWARD CHILDHOOD FEVER STUDY

Introduction: Fever is an abnormal rise in body temperature as part of a biological response regulated by the central nervous system. A body temperature above 37°C is considered fever. An axillary temperature of 37–37.5°C is classified as mild fever; 38–38.5°C as moderate fever; 38.5–39.5°C as high fever; and above 40°C as very high fever. Fever is one of the most common reasons for emergency department visits by children. Studies report that 20–30% of children presenting to emergency rooms globally, and up to 71% in our country, are due to fever. Many parents, however, lack essential knowledge for managing fever in children. Families across different cultures have unique beliefs and practices regarding fever. Some seek medical attention immediately, while others use traditional home remedies. Parental fear of fever contributes to excessive use of antipyretics and antibiotics, sometimes resulting in harmful outcomes.

This study aims to understand parental anxiety, knowledge, and practices regarding childhood fever. **Your participation will contribute to scientific knowledge. The survey takes approximately 3 minutes to complete. No compensation is offered**.

If you have any questions, please contact Dr. Kazım Kutlutürk at +90 505 923 16 95.

Section 1: Demographics

---

1. Your age: ……………………………………..

2. Number of children: ……………………………………..

3. Does your child have a chronic illness?

☐Yes

☐No

4. At what age did you become a mother?: ………………………………………..

5. What is your highest level of education?

☐Primary

☐Secondary

☐High school

☐University or higher

6. Your occupation:

☐Housewife

☐Civil servant

☐Worker

☐Teacher

☐Healthcare worker

☐Other

7. What is your monthly household income?

☐Below 5,500 TL

☐5,500–10,000 TL

☐Above 10,000 TL

Section 2: Fever Management

---

8. How do you usually measure your child's temperature at home?

☐By touch

☐Classic (mercury) thermometer

☐Digital thermometer

9. Which site do you typically use for temperature measurement?

☐Axilla

☐Ear

☐Rectal

☐Oral

☐Other

10. At what temperature and over do you consider your child to have a fever?

☐36.5°C and over

☐37°C and over

☐37.5°C and over

☐38°C and over

☐38.5°C and over

☐39°C and over

11. What is your first response when your child has a fever?

☐Remove clothing

☐Sponge with vinegar

☐Bathe with cold water

☐Wipe with cologne

☐Lukewarm bath

☐Give antipyretic

☐Give antibiotic

12. Which complication of fever concerns you the most?

☐Febrile seizures

☐Brain damage

☐Coma

☐Dehydration

☐Serious illness

☐Death

☐None

Section 3: Medication Use

---

13. Do you believe antibiotics can reduce fever?

☐Yes

☐No

14. Which medications do you use to reduce fever?

☐Paracetamol

☐Ibuprofen

☐Aspirin

☐Corticosteroids

☐Antibiotics

☐Novalgina

15. How frequently do you give those medications, especially ibuprofen?

☐Every 1 hour

☐Every 2 hours

☐Every 3 hours

☐Every 4 hours

☐Every 6 hours

16. How frequently do you give those medications, especially paracetamol?

☐Every 1 hour

☐Every 2 hours

☐Every 3 hours

☐Every 4 hours

☐Every 6 hours

17. How do you administer antipyretics?

☐Oral

☐Rectal

18. Which route do you use most frequently?

☐Oral

☐Rectal

Section 4: Information and Practices

---

19. Where did you learn about fever management?

☐Doctor

☐Nurse or midwife

☐Friends or family

☐Internet, TV, or social media

20. How often do you check your child's temperature when they have a fever?

☐Every 15 minutes

☐Every 30 minutes

☐Every 60 minutes

☐Longer than 60 minutes

21. How do you determine the dose of antipyretics?

☐Using provided dosing device

☐Household spoon

☐Using a different drug's dosing device

22. Whose advice do you usually follow when giving antipyretics?

☐Doctor

☐Nurse

☐Pharmacist

☐Neighbor

☐Personal experience

23. Do you give antipyretics after vaccination?

☐Yes

☐No

24. Have you ever used antibiotics without a prescription for fever?

☐Yes

☐No

25. On a scale of 1 to 10, how anxious do you feel when your child has a fever?

…………………………..

End of the survey.

Thank you for your participation!
